# Monstrosity by Defect

**Published:** 1868-10

**Authors:** 


MONSTROSITY BY DEFECT.—While at the County Hospital some weeks since, we were shown by the resident physician, Dr. Garwood, a singular monster, to which one of the patients gave birth. The lower extremities, the pelvis, and all that portion of the body below the plane of the umbilicus, were perfectly developed and normal; but these parts constituted the child, if child it could be called. There was nothing more—all above the umbilicus was wanting. Evidently the embryo from its earliest conception had consisted of nothing more than the parts mentioned. The integument was intact and perfect, and closed over what existed of the abdominal cavity, in such a manner as showed that there had never been anything like amputation *in utero.* Not a trace of a scar was perceptible. At the same time with this monstrosity was born a child with imperforate anus and faulty development of one of its ears. Another most singular fact is, that of the twelve children which the mother has had, not one has been perfectly developed. We regret that circumstances have prevented our obtaining a more satisfactory history of this most interesting case. *Pacific Med. and Surg. Journal.—N. Y. Med. Journal.*

